# Laparoscopic and endoscopic cooperative surgery for leiomyosarcoma of the stomach: a case report with a review of the literature

**DOI:** 10.1186/s40792-021-01218-3

**Published:** 2021-06-18

**Authors:** Toru Takagi, Shin Saito, Shinichiro Yokota, Yuki Kaneko, Kazuya Takahashi, Rihito Kanamaru, Kentaro Kurashina, Yoshinori Hosoya, Joji Kitayama, Hirotoshi Kawata, Hiroyuki Osawa, Naohiro Sata

**Affiliations:** 1 Department of Surgery, Jichi Medical University, 3311-1 Yakushiji, Shimotsuke, Tochigi 329-0498 Japan; 2 Department of Surgery, Allegheny General Hospital, 320 East North Avenue, Pittsburgh, PA 1521 USA; 3 Department of Pathology, Jichi Medical University, Shimotsuke, Japan; 4 Department of Gastroenterology, Jichi Medical University, Shimotsuke, Japan

**Keywords:** Leiomyosarcoma of the stomach, Gastrointestinal stromal tumor, Submucosal tumor, Mesenchymal tumor, Laparoscopic and endoscopic cooperative surgery, Case report

## Abstract

**Background:**

Leiomyosarcoma is a rare tumor that could originate from the gastrointestinal tract, uterus, kidney, retroperitoneum, and the soft tissues of the extremities. It accounts for only 1% of all gastrointestinal mesenchymal tumors and primary leiomyosarcoma of the stomach is extremely rare. Most cases reported as leiomyosarcoma of the stomach before the development of KIT immunohistochemistry might be gastrointestinal stromal tumors (GISTs) of the stomach and only 18 cases of leiomyosarcoma of the stomach have been reported since early 2000s. We report here a patient with leiomyosarcoma of the stomach treated by laparoscopic and endoscopic cooperative surgery (LECS).

**Case presentation:**

A 59-year-old man was referred to our hospital for an early gastric cancer, which was initially treated by endoscopic submucosal dissection. Six months after his initial treatment, a follow-up esophagogastroduodenoscopy revealed a small polypoid lesion at the lesser curvature of the proximal stomach, which appeared to be a hyperplastic polyp. However, one and a half years later, the lesion grew and showed more irregular surface. Biopsy at the time revealed smooth muscle cell proliferation suggestive of leiomyoma. Three years later, the lesion grew even larger and biopsy showed pleomorphic spindle cells. Immunohistochemical study showed positive staining for alpha-smooth muscle actin and desmin, but negative for c-kit and CD34. Ki-67 labeling index was nearly 60%. Based on these findings, the diagnosis of leiomyosarcoma was established. The patient subsequently underwent a partial gastrectomy by LECS. The patient is currently in good condition without recurrence or metastasis at 12 months after surgery.

**Conclusions:**

Leiomyosarcoma of the stomach is extremely rare. This is the first report of leiomyosarcoma of the stomach treated by LECS. We could also follow its appearance change through endoscopic examination for 3 years.

## Background

Leiomyosarcoma is a rare tumor that could originate from the gastrointestinal tract, uterus, kidney, retroperitoneum, and the soft tissues of the extremities [1]. Primary leiomyosarcoma of the stomach is extremely rare, and most cases reported as leiomyosarcoma of the stomach before the development of KIT immunohistochemistry (pre-KIT era) might be gastrointestinal stromal tumors (GISTs) of the stomach [2]. To the best of our knowledge, only 18 patients with leiomyosarcoma of the stomach have been described in the literature. Currently, the standard treatment is surgical resection of the tumor [2]. Herein, we report a patient with extremely rare leiomyosarcoma of the stomach. We observed the growth of this tumor endoscopically for 3 years and treated the tumor with laparoscopic and endoscopic cooperative surgery (LECS). This is the first report of leiomyosarcoma of the stomach treated by LECS.

## Case presentation

**Fig. 1:**
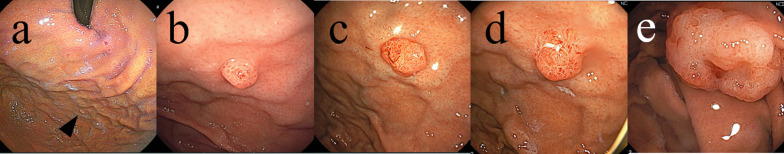
EGD showed a polypoid lesion at the lesser curvature of the proximal stomach. The lesion became larger. **a** A half year, **b** a year, **c** one and a half years, **d** 2 years, **e** 3 years from endoscopic submucosal dissection for an early gastric cancer

A 59-year-old male without significant chief complaints and medical history was referred to our hospital for treatment of early gastric adenocarcinoma. The patient underwent endoscopic submucosal dissection (ESD) to resect the lesion. Pathological examination demonstrated complete resection of the tumor. The resected tumor was confined within the mucosal layer without lymphovascular invasion. Six months after ESD, a follow-up esophagogastroduodenoscopy (EGD) was performed and found a small polypoid lesion at the lesser curvature of the proximal stomach, which appeared to be a benign polyp (Fig. 1a).

Subsequent EGD at 1 year after ESD, the polypoid lesion was clinically diagnosed as hyperplastic polyp based on its size (3 mm) and shape (Fig. 1b). One and a half years after ESD, the polypoid lesion grew in size and its surface became more irregular (Fig. 1c). However, biopsy did not show any signs of malignancy. Two years after initial ESD, biopsy showed smooth muscle cell proliferation without positive staining for the GISTs makers such as kit and CD34, which suggestive of leiomyoma (Fig. 1d). Three years after ESD, the lesion became even larger (8 mm in size) and its surface and vascular pattern became more irregular (Fig. 1e). Biopsy at this point revealed the tumor comprised pleomorphic spindle cells with noticeable atypia. Immunohistochemical study demonstrated positive staining for alpha-smooth muscle actin and desmin, but negative for c-kit, CD34, and AE1/AE3. Ki-67 labeling index was estimated as 60% and p16 was diffusely positive. Based on these findings, the tumor was diagnosed as leiomyosarcoma of the stomach. Computed tomography scans and fluorodeoxyglucose positron emission did not show any distant metastatic disease.

**Fig. 2:**
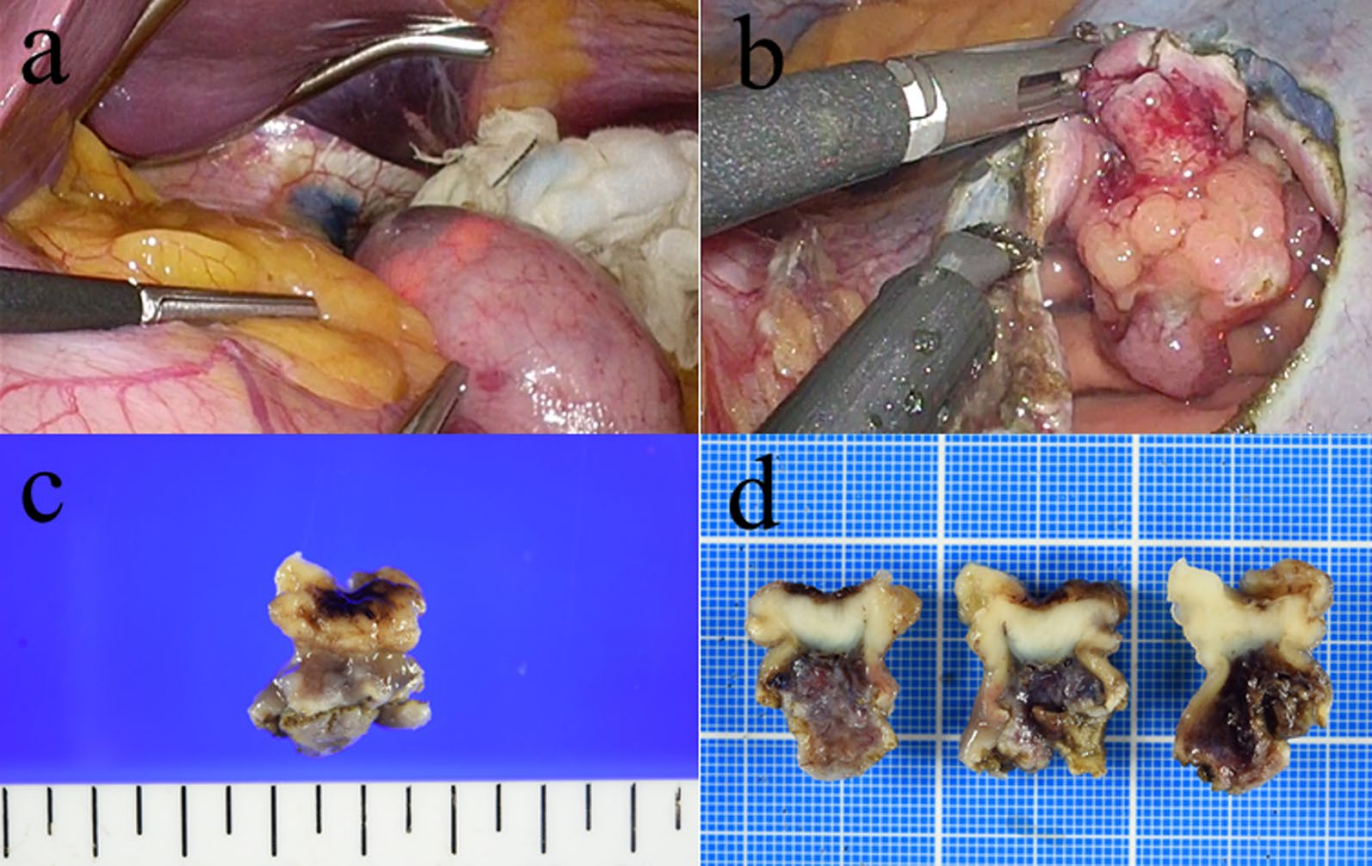
Intraoperative findings and resected specimen. The periphery of the tumor was marked by injecting indigo carmine and hyaluronic acid into the submucosal layer (**a**); the stomach was dissected along the marked incision line laparoscopically (**b**); macroscopically, the tumor measured 18 × 15 × 10 mm in size (**c**); relatively well-circumscribed, solid white tumor (**d**)

The patient subsequently underwent a partial gastrectomy by LECS. The location of the tumor was confirmed by intraoperative EGD, but was not identifiable with laparoscopy. The margin of the tumor was marked endoscopically by injecting both indigo carmine and hyaluronic acid into the submucosal layer (Fig. 2a). The gastric wall was dissected along the incision line from the laparoscopic side (Fig. 2b). The dissection line was clearly identified both laparoscopically and endoscopically. After the resection of the lesion, the incision was closed using hand-sewn sutures laparoscopically.

**Fig. 3:**
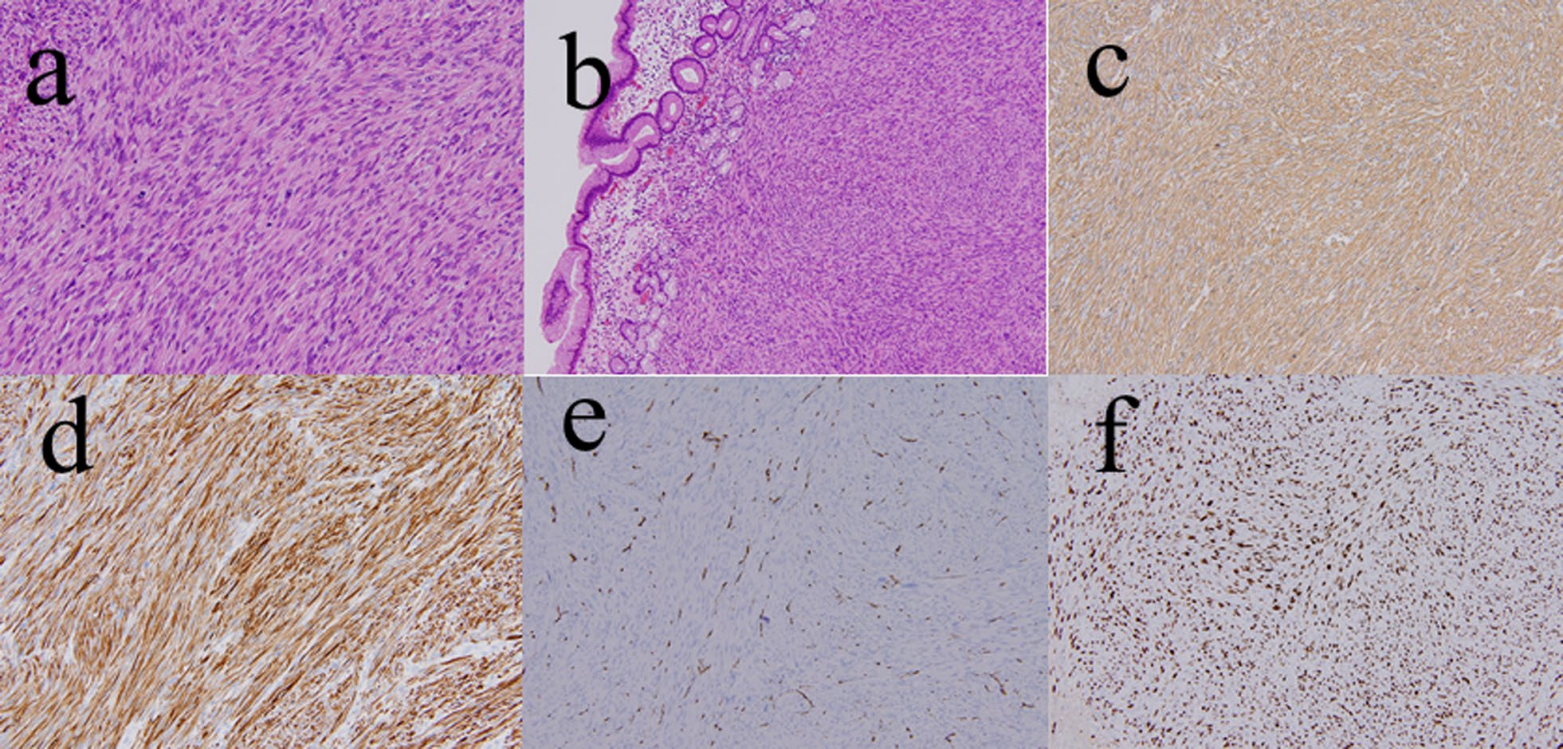
Pathological findings. Hematoxylin and eosin staining shows well-developed fascicles of atypical spindle cells with eosinophilic cytoplasm (**a**) (200×); derived from proper mucous membrane and submucosa (**b**) (100×). Immunohistochemistry showed the tumor cells were positive for alpha-smooth muscle actin (**c**) (200×), and desmin (**d**) (200×), and negative for CD 34 (**e**) (100×). Ki-67 index was estimated to be 60% (**f**) (100×)

Macroscopically, the lesion was a well-circumscribed, solid white tumor and measured 18 × 15 × 10 mm in size (Fig. 2c, d). Histologically, hematoxylin and eosin staining revealed that the tumor was composed of atypical spindle cells with eosinophilic cytoplasm (Fig. 3a) derived from proper mucous membrane and submucosa (Fig. 3b). Immunohistochemistry demonstrated that the tumor cells were positive for alpha-smooth muscle antin (Fig. 3c) and desmin (Fig. 3d), but negative for CD34 (Fig. 3e) and S-100 protein, which was consistent with preoperative biopsy specimens. Ki-67 index was estimated to be 60% (Fig. 3f). Based on these findings, the diagnosis of leiomyosarcoma was confirmed. Post-operative course was uneventful and the patient was discharged on postoperative day 9. The patient underwent computed tomography scans every 6 months and EGD annually for postoperative surveillance. The patient also had blood work done every 3 months. The patient is currently in good condition without recurrence or metastasis at 12 months after surgery.

## Discussion

Leiomyosarcoma is a malignant mesenchymal tumor arising from smooth muscle cells [3]. Leiomyosarcoma of the gastrointestinal tract is a rare entity, which accounts for 1.1% of all gastrointestinal mesenchymal lesions [4]. Moreover, the stomach is the least common site for true leiomyosarcoma to originate [5]. Thus, gastric leiomyosarcoma is extremely rare. Leiomyosarcoma shows the pattern of intersecting marginated fascicles of spindle cells microscopically [6]. GISTs are also mesenchymal tumors with malignant potential [7]. GISTs show spindle cell pattern microscopically, which are believed to arise from the interstitial cells of Cajal or their precursors, located throughout the muscular wall of the gastrointestinal tract [8]. Immunohistochemistry allows clinicians to differentiate leiomyosarcoma from GISTs. Leiomyosarcoma usually lacks staining for CD117, CD34 and kit [9] which are positive in the majority of GISTs [10]. By contrast, leiomyosarcoma is usually positive for smooth muscle cell markers such as actin, desmin, and h-caldesmon [9].

Most cases reported in the “pre-KIT era” as leiomyosarcoma of the stomach might be GISTs [2]. Only 19 cases of leiomyosarcoma of the stomach have been reported since early 2000s (Table 1), including our patient in this report. Patients included 11 males and 8 females. Their age ranged between 16 and 74 years with median of 51. Leiomyosarcomas of the stomach are likely to present as polypoid lesion rather than submucosal tumor or ulcerated lesion. In our case, the lesion exhibited polypoid appearance and showed progressive growth. In contrast, gastric leiomyomas and GISTs usually show submucosal tumor appearance [24]. Thus, the difference in endoscopic appearances may be the clue to discriminate leiomyosarcoma from leiomyomas and GISTs.

**Table 1:** Cases of leiomyosarcoma of the stomach reported since early 2000s

Case no	Age/sex	Location	Size (cm)	Endoscopic appearance	Histological location	Preoperative diagnoses	Treatment	Outcome (F/U period)	References
1	74/F	Upper	1.5	Polypoid lesion	Intramucosa to submucosa	−	Endoscopic resection	Alive (36 months)	[1]
2	72/F	Middle	2.5	Polypoid lesion	Muscularis mucosae	N, M	Endoscopic resection	N, M	[5]
3	37/M	Lower	1	Polypoid lesion	N, M	−	Endoscopic resection	Alive (3 years)	[11]
4	63/F	Upper	9	Polypoid lesion	N, M	−	Laparotomy	N, M	[12]
5	47/M	Middle	13 × 13 × 10	N, M	N, M	N, M	Laparotomy	Alive (35 months)	[13]
6	51/M	Upper	4 × 1.6	Ulcerated lesion	Mucosa, submucosa and muscularis propria	−	Laparotomy	Alive (10 months)	[14]
7	16/F	Upper	N, M	Submucosal tumor	N, M	−	Laparotomy	Alive (18 months)	[15]
8	25/M	Middle	18 × 12 × 7	N, M	Muscularis propria	−	Laparotomy	Alive (1 year)	[16]
9	57/F	Middle	13 × 13 × 5	N, M	N, M	−	Surgical resection (details unknown)	DUC (1 year)	[2]
10	51/M	N, M	2.5	Submucosal tumor	Mucosa to submucosa	N, M	Surgical resection (details unknown)	Alive (18 months)	[4]
11	74/M	N, M	8	N, M	N, M	N, M	Surgical resection (details unknown)	DUC (9 years)	[9]
12	71/M	Middle	9 × 8 × 3	Ulcerated lesion	unknown	unknown	Surgical resection (details unknown)	Alive (28 months)	[17]
13	29/F	Upper	11 × 9.7 × 3.2	Polypoid lesion	Muscularis propria	−	Surgical resection (details unknown)	Alive (8 months)	[18]
14	65/M	N, M	8.5	N, M	N, M	N, M	Surgical resection (details unknown)	DOD (2 years)	[19]
15	26/M	Upper	7.2	N, M	Muscularis mucosae	N, M	Surgical resection (details unknown)	Alive (1 month)	[20]
16	48/M	Unknown	N, M	N, M	N, M	**+**	Surgical resection (details unknown)	Alive (58 months)	[21]
17	43/F	N, M	3	N, M	N, M	−	Surgical resection (details unknown)	DOD (20 months)	[22]
18	48/F	Middle	2	Polypoid lesion	N, M	+	Chemotherapy	DOD (1 year)	[23]
19	59/M	Upper	1.8 × 1.5 × 1	Polypoid lesion	Muscularis propria and submucosa	+	Laparoscopic/endoscopic cooperative surgery	Alive (1 year)	Our case

*M* male, *F* female, *N, M* not mentioned, *F/U* follow-up, *DOD* dead of disease, *DUC* dead of unknown cause

We happened to observe the lesion for 3 years after initial resection by ESD, because we initially thought it was a small benign polyp and our first and second biopsies failed to establish accurate diagnosis to the lesion.

Diagnosis of leiomyosarcomas relies on histopathological examination of biopsies from deeper layer because these arise between the muscularis propria and muscularis mucosa layers [2]. Conventional endoscopy usually yields superficial and normal mucosa [12]. Simple endoscopic biopsy specimens may be insufficient to establish the diagnosis due to the overlying mucosa [25]. Sampling error maybe the reason why we initially misdiagnosed the polypoid lesion as a benign polyp. Fine-needle aspiration obtained with endoscopic ultrasound guidance may be a more accurate alternative method to establish the pathological diagnosis of a submucosal tumor. We should have considered this method for this patient in an earlier stage.

Sato et al. reported the first case of primary leiomyosarcoma of the stomach that was successfully resected by ESD as a diagnostic method [1]. Pauser et al. also reported endoscopic resection of leiomyosarcoma of the stomach that was unexpectedly diagnosed because they initially suspected the lesion to be a hyperplastic polyp or an adenoma [11]. However, ESD for mesenchymal tumors in gastrointestinal tracts might lead to incomplete resection since they could originate from deeper layer of the intestinal wall [2].

The standard treatment for leiomyosarcoma is complete surgical resection of the tumor because it has malignant potential and does not respond to targeted treatment with tyrosine kinase inhibitor [1]. Previous reports have suggested that lymph node dissection is not recommended for gastric mesenchymal tumors including GISTs since most tumors rarely metastasize to regional lymph nodes [26]. However, tumors should be resected without the capsule damage because the tumor rupture is classified to high-risk group for the recurrence [27]. LECS is a useful approach that allows endoscopic evaluation of an intra-luminal mass such as gastric submucosal tumors and is less invasive than laparotomy [28]. We successfully completed LECS to treat leiomyosarcoma of the stomach. To the best of our knowledge, this is the first report of leiomyosarcoma of the stomach treated by this method. LECS is a procedure combining laparoscopic gastric resection with ESD for local resection of gastric tumors with appropriate minimal surgical resection margins [29]. Most of gastric mesenchymal tumors are recognized intra-luminally on the mucosal side and these tumors sometimes cannot be visualized laparoscopically from the outside of the stomach. It could be difficult to determine accurate resection lines for gastric submucosal tumors using conventional laparoscopic partial resection. The inappropriate resection lines may result in the positive resection margins. Therefore, endoscopic determination of an accurate cutting line is critical for the complete resection of tumors in the stomach [29]. In our case, the tumor margin was marked by injecting indigo carmine and hyaluronic acid into the submucosal layer. The stomach wall was then dissected along the marked incision line laparoscopically and we could obtain the appropriate margin.

Recently, non-exposed endoscopic wall-inversion surgery (NEWS) or combination of laparoscopic and endoscopic approaches to neoplasia with non-exposure techniques (CLEAN-NET) were developed as a full-thickness resection without intestinal perforation [29]. NEWS or CLEAN-NET are alternative methods to resect tumor while preventing tumor cell from seeding into the abdominal cavity. Based on the pathological findings of biopsy specimen, the lesion was thought to be mesenchymal lesion, suggesting it was covered with normal mucosa.

## So, we adapted LECS to the lesion and the lesion was histopathologically covered with normal mucosa (Fig. 3b).

Our report indicates that mesenchymal tumors of the stomach including leiomyosarcoma can be difficult to establish the diagnosis from conventional endoscopic biopsy specimens. LECS can be useful to establish the pathological diagnosis and simultaneously allow a curative resection of the gastric submucosal tumor.

## Conclusion

Leiomyosarcoma of the stomach is extremely rare. This is the first report of leiomyosarcoma of the stomach treated by LECS. We could also follow its appearance change through endoscopic examination for 3 years.

## Data Availability

Not applicable.

## Abbreviations

ESD: Endoscopic submucosal dissection
GIST: Gastrointestinal stromal tumor
LECS: Laparoscopic and endoscopic cooperative surgery
EGD: Esophagogastroduodenoscopy
NEWS: Non-exposed endoscopic wall-inversion surgery
CLEAN-NET: Combination of laparoscopic and endoscopic approaches to neoplasia with non-exposure techniques
